# Mechanism of Resistance to *S*-metolachlor in Palmer amaranth

**DOI:** 10.3389/fpls.2021.652581

**Published:** 2021-03-12

**Authors:** Gulab Rangani, Matheus Noguera, Reiofeli Salas-Perez, Lariza Benedetti, Nilda Roma-Burgos

**Affiliations:** ^1^Department of Crop, Soil and Environmental Sciences, University of Arkansas, Fayetteville, AR, United States; ^2^Crop Protection Graduate Program (Programa de Pós-Graduação em Fitossanidade), Federal University of Pelotas (Universidade Federal de Pelotas), Pelotas, Brazil

**Keywords:** Palmer amaranth, S-metolachlor, tolerance, resistance, GST, gene expression, NTSR

## Abstract

Herbicides are major tools for effective weed management. The evolution of resistance to herbicides in weedy species, especially contributed by non-target-site-based resistance (NTSR) is a worrisome issue in crop production globally. Glyphosate-resistant Palmer amaranth (*Amaranthus palmeri*) is one of the extremely difficult weeds in southern US crop production. In this study, we present the level and molecular basis of resistance to the chloroacetamide herbicide, *S*-metolachlor, in six field-evolved *A. palmeri* populations that had survivors at the recommended field-dose (1.1 kg ai ha^−1^). These samples were collected in 2014 and 2015. The level of resistance was determined in dose-response assays. The effective dose for 50% control (ED_50_) of the susceptible population was 27 g ai ha^−1^, whereas the ED_50_ of the resistant populations ranged from 88 to 785 g ai ha^−1^. Therefore, *A. palmeri* resistance to *S*-metolachlor evolved in Arkansas as early as 2014. Metabolic-inhibitor and molecular assays indicated NTSR in these populations, mainly driven by GSTs. To understand the mechanism of resistance, selected candidate genes were analyzed in leaves and roots of survivors (with 1 × *S*-metolachlor). Expression analysis of the candidate genes showed that the primary site of *S*-metolachlor detoxification in *A. palmeri* is in the roots. Two *GST* genes, *ApGSTU19* and *ApGSTF8* were constitutively highly expressed in roots of all plants across all resistant populations tested. The expression of both *GST*s increased further in survivors after treatment with *S*-metolachlor. The induction level of *ApGSTF2* and *ApGSTF2like* by *S*-metolachlor differed among resistant populations. Overall, higher expression of *ApGSTU19, ApGSTF8, ApGSTF2*, and *ApGSTF2like*, which would lead to higher GST activity in roots, was strongly associated with the resistant phenotype. Phylogenetic relationship and analysis of substrate binding site of candidate genes suggested functional similarities with known metolachlor-detoxifying GSTs, effecting metabolic resistance to *S*-metolachlor in *A. palmeri*. Resistance is achieved by elevated baseline expression of these genes and further induction by *S*-metolachlor in resistant plants.

## Introduction

The evolution of resistance in response to intensive herbicide selection pressure in weedy species is a worldwide problem in crop production. Understanding the underlying physiological and molecular basis of resistance evolution is useful in long-term resistance management and design of new agrichemicals. *S*-metolachlor is a member of the chloroacetamide chemical family under Group 15 of the Weed Science Society of America (WSSA) classification system. Group 15 herbicides are soil-active and inhibit seedling root and shoot growth by blocking the formation of very long chain fatty acids (VLCFA, site-of-action), acting even before the susceptible grass or broadleaf weeds emerge. *S*-metolachlor is typically used preemergence in corn (*Zea mays* L.), cotton (*Gossypium hirsutum* L.), soybean [*Glycine max* (L.) Merr] and many other crops, including turfgrass, for the control of grasses and small-seeded broadleaf weeds. Among the primary weeds controlled by *S*-metolachlor are *Amaranthus* species. In the USA, *S*-metolachlor is the third largest volume of herbicide active ingredient used, following glyphosate and atrazine (Atwood and Paisley-Jones, 2017).

Target-site and non-target-site-based resistance (NTSR) mechanisms are the evolved physiological, molecular or genetic changes in weed populations that allow them to survive, or escape, herbicide application (Jugulam and Shyam, 2019; Gaines et al., 2020). Target-site resistance is endowed mostly by modification or amplification of the target enzyme, involves single gene, and does not contribute to multiple resistance with other herbicide sites of action (SOA). NTSR includes reduced translocation, sequestration and/or metabolic degradation of herbicide to non-toxic metabolite(s), among other plant modifications. The most adverse effect of metabolism-based resistance is multiple resistance to herbicides with different SOAs, or resistance to new herbicide chemistries yet “unseen” by weeds. This is a serious threat to weed management as NTSR can eliminate the utility of many herbicide tools, which threatens global food security. Metabolic degradation of herbicides is complex. It is generally facilitated by inherited or elicited molecular responses, which involve multiple superfamily genes such as cytochrome (P450) monooxygenase and glutathione-S-transferase (GST). A similar type of enhanced molecular response is also elicited using safeners, formerly referred to as herbicide antidotes, that can increase the detoxification rates of herbicides in cereal plants (Riechers et al., 2010). Safeners are used to increase crop tolerance to certain herbicide chemistries such as chloroacetanilides (Davies and Caseley, 1999). Safeners reduce the phytotoxicity of chloroacetamides to corn, sorghum, wheat, rice and barley by inducing specific GSTs, which are the main detoxifying agents for chloroacetamides and are involved in metabolic detoxification of certain other herbicides (Cummins et al., 1997; Dixon et al., 1997; Gronwald and Plaisance, 1998; Pascal and Scalla, 1999; Wu et al., 1999; Deng and Hatzios, 2002a,b; Scalla and Roulet, 2002). Although the use of safeners is based on selective action on cereal crops (Zhang and Riechers, 2004), apparent conservation in molecular response involving safener recognition and GST induction in Arabidopsis has been observed (DeRidder et al., 2002; Edwards et al., 2005; DeRidder and Goldsbrough, 2006; Riechers et al., 2010).

Palmer amaranth *(Amaranthus. palmeri* S. Wats) is now one of the major, and most difficult, weeds to control in corn, cotton, and soybean production. Resistance to glyphosate and acetolactate synthase (ALS) inhibitors among *A. palmeri* populations is rampant. *A. palmeri* has evolved resistance to eight herbicide SOAs including that of protoporphyrinogen oxidase (PPO)-inhibitors (Noguera et al., 2020) and *S*-metolachlor (Brabham et al., 2019) in the mid-southern US, starting in Arkansas. The increase in resistance to PPO inhibitors forced farmers to rely more on VLCFA inhibitors for *A. palmeri* control, further reducing the diversity of herbicides and spectrum of control. Pre-existing NTSR to ALS- or PPO-inhibitors could also have increased the likelihood of resistance evolution to VLCFA inhibitors. In any case, this latest scenario is highly worrisome because the PPO inhibitors and VLCFA inhibitors, such as *S*-metolachlor, are the remaining pillars of chemical weed management for *A. palmeri* in various crops.

Target-site modification is an unlikely mechanism for tolerance or resistance to VLCFA inhibitors in crops and weedy species due to the multiple SOAs of different enzymes involved in VLCFA synthesis (Busi, 2014). Crop selectivity to several chloroacetamide herbicides and safeners is mediated by enhanced GST activity, as a result of increased *GST* expression (Leavitt and Penner, 1979; Lamoureux and Rusness, 1989; Frova, 2006; Riechers et al., 2010). Enhanced amount of GSTF1 protein, a biomarker of NTST was found in *L. rigidum* population that showed reduced sensitivity to VLCFA inhibitors Torra et al. (2021). Thus far, resistance to VLCFA inhibitors in weedy species is attributed to NTSR mechanism mediated by enhanced GST activity (Busi et al., 2018; Brabham et al., 2019; Dücker et al., 2020). GSTs from the phi (GSTF) and tau (GSTU) classes are unique to plants and its role has been widely investigated in stress tolerance and secondary metabolism as well as in detoxification of herbicides in crops and weeds (Hatton et al., 1996; Cummins et al., 2011). GSTs catalyze the conjugation of glutathione (GSH) with a wide Hatton et al., range of endogenous and xenobiotic molecules and protect against oxidative damage. GSTFs and GSTUs show specificity toward different substrates. Phi enzymes are highly reactive toward chloroacetanilide and thiocarbamate herbicides. Some Phi GSTs have other functions including transport of flavonoid pigments to the vacuole, shoot regeneration and GSH peroxidase activity. Tau enzymes are highly efficient in detoxifying diphenylether and aryloxyphenoxypropionate herbicides. In addition, Tau GSTs have important roles in intracellular signaling, vacuolar deposition of anthocyanin, responses to soil stresses, auxin and cytokinin hormones (Edwards et al., 2005).

In this study we determined the *S*-metolachlor resistance level in six *A. palmeri* populations, examined the expression profile of candidate *GST* genes in these resistant populations in response to *S*-metolachlor, and provided evidence for its association with herbicide detoxification.

## Materials and Methods

### Plant Material

A late-season collection of *A. palmeri* inflorescences was done in the 2014–2016 summer(s) following established methodology (Burgos et al., 2013). Six populations from four Arkansas counties were included in this study, which will hereby be identified as: 15CRI-A, 14CRI-C, 14CRI-G, 14MIS-E, 14MIS-H, and 16WOO-A. A susceptible standard (SS) collected from Crawford, AR, was also included.

### Dose Response of *Amaranthus palmeri* Populations to *S*-metolachlor

To determine the resistance level to *S*-metolachlor, a dose-response experiment was conducted in the greenhouse. Fifty seeds of each population were sown in 400-mL pots filled with a 4:1 mixture of field soil:commercial potting medium and sprayed with the herbicide. Soil (Roxana silt loam) was collected at the Vegetable Research Station in Kibler, AR and mixed with Sunshine® Premix #1 (Sun Gro Horticulture, Bellevue, WA). The final physico-chemical properties of the mixture were: pH = 6.4, organic matter = 1.79%, and clay content = 15.1%. The experiment was conducted in a completely randomized design with three replications, with pot as experimental unit. The seeds were spread on the soil surface, then covered with a thin layer of the same soil mixture, and sprayed with eight rates of *S*-metolachlor (Dual II Magnum; Syngenta Crop Protection, LLC, Greensboro, NC) as follows: 0, 1/16x, 1/8x, 1/4x, 1/2x, 1x, 2x, and 3x, where x is the labeled dose for a silt loam soil (1.12 kg ai ha^−1^). For the SS, the lowest dose was 1/32x and the highest was 2x. Herbicide application was done in a spray chamber equipped with a motorized boom fitted with flat-fan 1100067 nozzles (Teejet, Wheaton, IL), calibrated to deliver 187 L ha^−1^ at 276 kPa. After application, the herbicide was activated by gently misting water on the soil surface. The pots were then placed in a greenhouse kept at 32/28C day/night temperature and 16-h day length. Throughout the study, soil in the pots were watered by capillarity as needed. At 28 days after application (DAT), seedlings were counted and survival (%) was calculated relative to the non-treated checks. Regression analysis was done with the packages *drc* and *mselect* in R 4.0.3 (Ritz et al., 2015). The appropriate model was selected based on the Akaike's information criterion and *p*-value for the lack-of-fit test (Ritz, 2010). Data from the accessions CRI-G and MIS-E were fitted with a three-parameter Log-logistic model (Eq. 1, with *c* = 0); WOO-A, CRI-A and SS, with a three-parameter Weibull II model (Eq. 3, with *c* = 0); CRI-C with a four-parameter Weibull II model (Eq. 3); and MIS-H with a three-parameter Weibull I model (Eq. 2).

(1) $$\begin{array}{r} Y=c+\frac{d-c}{1+\exp\left(b\left(\log\left(x\right)-\log\left(e\right)\right)\right)} \end{array}$$

(2) $$\begin{array}{r} Y=d(exp\left(-\exp\left(b\left(\log\left(x\right)-\log\left(e\right)\right)\right)\right) \end{array}$$

(3) $$\begin{array}{r} Y=c+\left(d-c\right)\left(1-\exp\left(-\exp\left(b\left(\log\left(x\right)-\log\left(e\right)\right)\right)\right)\right) \end{array}$$

In the equations above, *Y* is the survival percentage, *d* is the asymptote at the upper limit, *c* is the asymptote at the lower limit, *X* is the *S*-metolachlor rate and *b* is the slope around *e*, which is the value of *X* that causes a 50% reduction of *Y*. The ED50 of each accession was estimated and used for the determination of resistance level (ED50 R/ED50 SS).

### Herbicide Metabolism Inhibition by NBD-Cl

To verify the contribution of GSTs toward *S*-metolachlor resistance in *A. palmeri*, three populations (14CRI-G, 15CRI-A and SS) were tested in an agar-based plate assay with a GST inhibitor. The growth medium was prepared by dissolving 2.2 g of Murashige and Skoog basal salt mix (PhytoTech Labs Inc., Lenexa, KS) and 4 g of agar (Himedia Labs, West Chester, PA) in 500 mL deionized water. The pH was adjusted to 6.3 and the mixture was autoclaved. Analytical grade *S*-metolachlor (Sigma-Aldrich Inc., St. Louis, MO) and the GST inhibitor 4-chloro-7-nitrobenzofurazan (NBD-Cl; Sigma-Aldrich) were dissolved and diluted in chloroform. Work solutions were prepared through serial dilutions to achieve *S*-metolachlor concentrations of 0, 0.06, 0.12, 0.25, 0.5, 1, 2, 4, and 8 μM, and NBD-Cl concentrations of 0 and 25 μM in the growth medium. Herbicide and inhibitor rates were chosen based on preliminary experiments (data not shown). A final chloroform concentration of 0.14% was kept constant in all plates by adding 24 μL of each work solution to 30 mL of growth medium to each plate. The plates were sterile, square, gridded Petri dishes (100 × 100 × 15 mm, 13-mm grids, Simport Scientific Inc., Beloeil, QB, Canada). Each plate had 30 seeds. The plates were sealed with Parafilm tape (Bemis Company Inc, Neenah, WI) and kept in a growth chamber at 30/28°C day/night temperature, and 16-h daylight. At 14 DAT, the plates were photographed and root lengths were measured using ImageJ (Schneider et al., 2012). The square grids were used as scale for unit conversion. From each plate, 10–15 representative roots were measured, which were considered biological replicates. The experiment was repeated and data from both runs were pooled as results across runs did not differ statistically. Root length data were fitted to a four-parameter, log logistic model using the package *drc* in R 4.0.3, as defined previously in Eq. 1. To determine the sole effect of NBD-Cl in the absence of herbicide, a subset of the data was submitted to ANOVA and means were compared using a Tukey's HSD test in the *multcomp* package in R.

### Selection of Candidate Genes

Homologs of known *GST* genes in *A. palmeri* were identified using BLAST tool from CoGe (https://genomevolution.org/coge/SearchResults.pl?s=amaranthus&p=genome). The top similar genes were identified as candidate genes. Additionally, NCBI BLAST tool was also used to examine the homology between all selected genes within selected species.

### Gene Expression Analysis

For candidate gene expression analysis, survivors of 1x field rate from the resistant (R) populations were sampled. Gene expression analysis was conducted using leaf and root tissues. Three biological replicates were used for leaf tissue analysis. Three to five leaf segments, ~0.5 cm long, were sampled from a single plant and 2–5 plants were pooled together from the same population. The leaf tissues from treated plants were harvested 21 days after *S*-metolachlor treatment. Two biological replicates of treated leaf tissues were composed of pooled tissues from at least 6–10 R plants per population and the third biological replicate was comprised of tissue collected from a single plant from each population. Leaves of non-treated control plants from R and S populations were sampled at 21 days after planting and all three biological replicates consisted of pooled plants. Roots were collected using the same pooled plants used for leaf tissue sampling. Equal amounts of root tissue from each treated plant (resistant survivor) were pooled to represent two biological replicates per population. Root tissue collected from a single plant was not enough to conduct the expression analysis of all genes; therefore, the third biological replicate, which is a single plant sampled for leaf tissue, could not be included in the root tissue analysis. Similarly, root tissues of non-treated plants were pooled representing three biological replicates from each population. Total RNA was isolated using an E.Z.N.A ® Plant RNA isolation kit (Omega Bio-tek, Norcross, GA) and converted to cDNA using RevertAid First Strand cDNA Synthesis Kit (Thermo Fisher Scientific, Waltham, MA, USA) according to manufacturer's instructions using 1 μg of total RNA for each sample. The differences in transcript abundance of five candidate genes; *ApGSTU18, ApGSTU19, ApGSTF8, ApGSTF2*, and *ApGSTF2like* were validated by RT-qPCR using iCycler Real-Time PCR Detection System (Bio-Rad Laboratories Inc.). Each qPCR reaction contained 1X IQ^TM^ SYBR Green Supermix (2x) (Bio-Rad Laboratories), 1 μl of cDNA (1:5 dilution), and 0.5 μM of gene-specific primers. Relative expression levels of each gene were calculated using the 2^−ΔΔCt^ algorithm (Livak and Schmittgen, 2001) by normalizing to the expression of ß*-tubulin* (Nakka et al., 2017) and *elongation factor1*α gene. Fold change in each resistant population was measured against non-treated S plants.

### Phylogenetic Analysis and Active-Site Comparison

A phylogenetic tree of GSTs from *A. palmeri, Zea mays, Arabidopsis thaliana, Lolium rigidum*, and *Alopecurus myosuroides* was constructed using standard configuration in MEGA-X. The H-site was added manually using NCBI CD-search tool in the candidate GST enzyme sequence. The full-length sequence of *ApGSTU19* was obtained using the primers listed in Supplementary Table 1. Total RNA of the two survivors from 14CRI-G to 15CRI-A population was converted to cDNA as described in the previous section. The PCR was conducted in a 20-μL volume that consisted of 1 μL cDNA, 0.5 μM of each primer, and 10 μL EmeraldAmp MAX PCR Master Mix (Takara). The PCR was run with the following profile: 98°C for 30 s; 30 cycles of 98°C for 30 s, 58°C for 30 s and 72°C for 1 min; followed by a final extension step of 10 min at 72°C. The PCR product was purified from agarose gel using GeneJET Gel Extraction Kit (Thermo Fisher Scientific) and sequenced by Psomagen Inc. (Rockville, MD). The resulting overlapping fragments were assembled into one sequence to get the complete coding region of *ApGSTU19* using Sequencher (Gene Codes Corporation, Ann Arbor, MI) software. The nucleotide sequences were translated into open reading frames using ExPASy's online translation tool, and compared to the reference using Uniprot Align tool.

## Results

### *S*-metolachlor Resistance Level in Field-Evolved Populations

**Figure 1:**
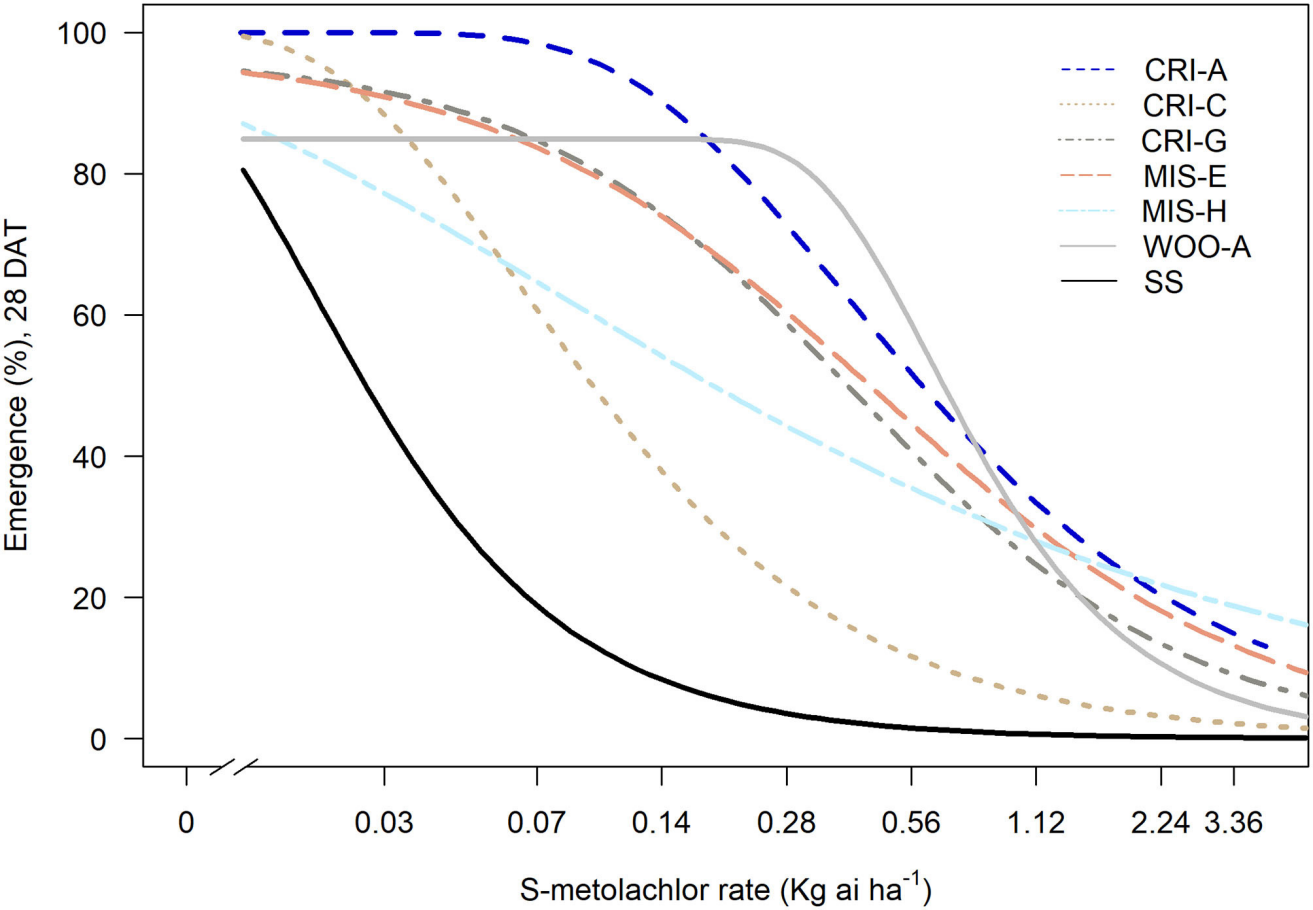
*Amaranthus palmeri* survival (%) relative to non-treated plants in response to *S*-metolachlor. SS is susceptible standard; all others are resistant.

In the general screening of 115 samples collected between 2014 and 2016 with 1x dose (1.12 kg ai ha^−1^) *S*-metolachlor, six populations showed reduced sensitivity to *S*-metolachlor, while the rest was controlled 100% (data not shown). The ED_50_ of susceptible population (SS) was 27 g ai ha^−1^, whereas the ED_50_ of the resistant populations ranged from 88 to 784 g ai ha^−1^ (Figure 1, Table 1). Thus, the levels of resistance to *S*-metolachlor in 16WOO-A, 15CRI-A, 14CRI-G, 14-MIS-H, 14CRI-C, and 14MIS-E ranged from 3- to 29-fold relative to the SS population (Table 1). Several resistant populations had survivors at the 2x dose and notably more survivors at the sublethal dose (0.5x) compared to the S population (Supplementary Figure 1).

**Table 1:** Resistance levels of S-metolachlor resistant *Amaranthus palmeri* populations.

**Population**	**ED_**50**_ (g ai ha^**−1**^)^a^**	**R/S^b^**
16WOO-A	785 (521–1049)	29
15CRI-A	593 (449–737)	22
14CRI-G	418 (244–592)	15
14CRI-C	88 (69–107)	3
14MIS-H	207 (37–376)	8
14MIS-E	467 (221–712)	17
SS	27 (21–33)	1

a *ED_50_, estimated dose of S-metolachlor herbicide required to cause 50% injury. Values in parenthesis are 95% confidence intervals*.

b *R/S, ED_50_ of the resistant population divided by the ED_50_ of the SS population*.

### Effect of GST Inhibitor and *S*-metolachlor on the Seedling Growth of *A. palmeri*

To verify that GST enzymes were involved in resistance to *S*-metolachlor, we tested the effect of *S*-metolachlor on seedling growth of *A. palmeri* in the presence and absence of GST inhibitor, NBD-Cl. Seeds of two resistant populations, 15CRI-A and 14CRI-G, and SS were first germinated in a medium containing a range of NBD-Cl concentrations (25 μM to 0.1 mM) to determine the maximum tolerated dose that would cause minimum injury (Data not shown). Symptoms of NBD-Cl phytotoxicity appeared in the form of stunted root and cotyledon growth even at the lowest concentration tested. The lowest concentration (25 μM) was used to study the inhibitor effect on *S*-metolachlor phytotoxicity.

**Figure 2:**
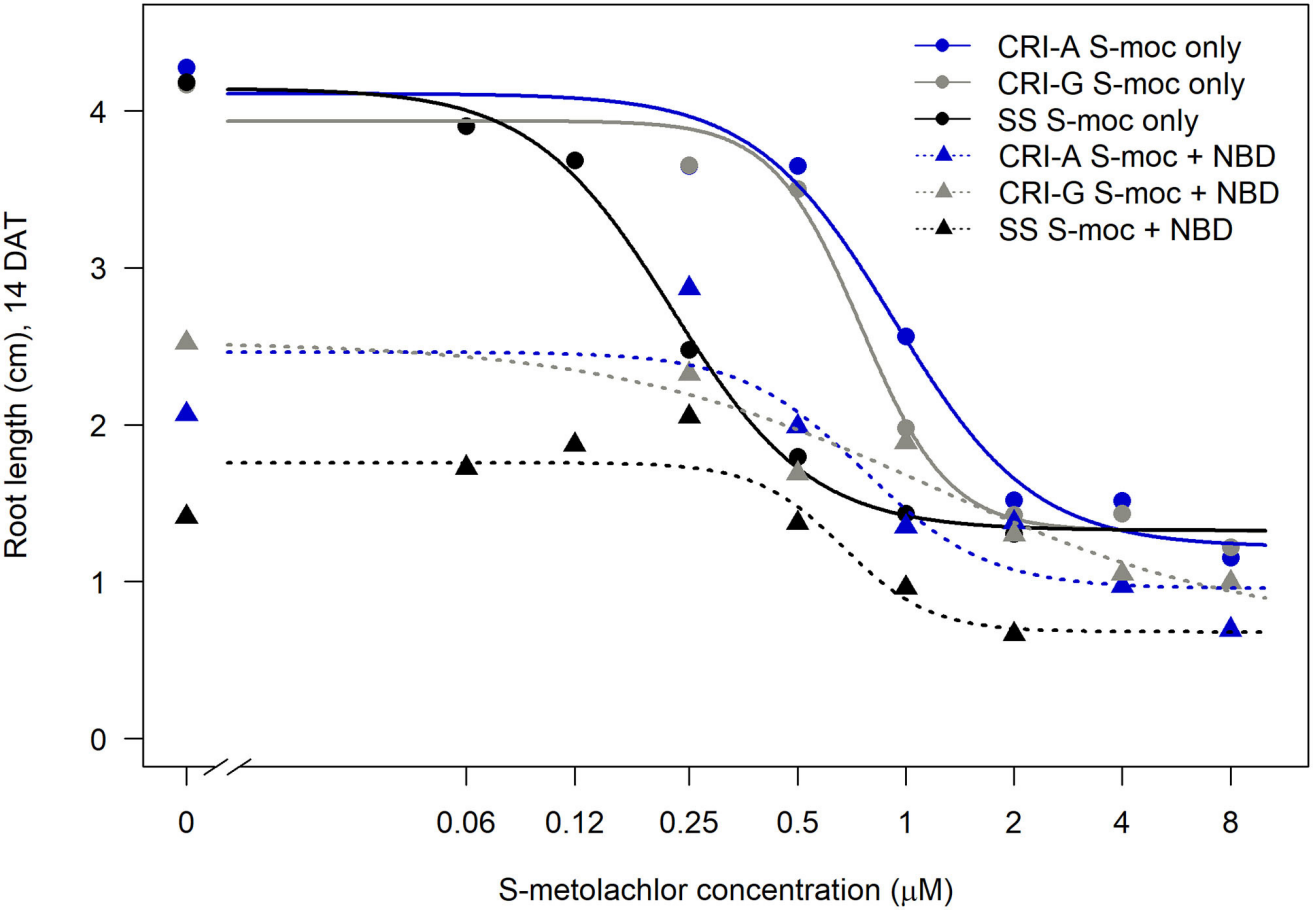
Effect of GST inhibitor, NBD-Cl, and *S*-metolachlor on root growth of *Amaranthus palmeri* in agar-based assays. 15CRI-A and 14CRI-G are resistant populations. NBD-Cl was used at 0.25 μM. Root lengths were measured at 14 days of incubation in a growth chamber set at 30/28°C day/night temperature. The experiment was conducted twice. Each data point is the average of 25–30 plants.

**Figure 3:**
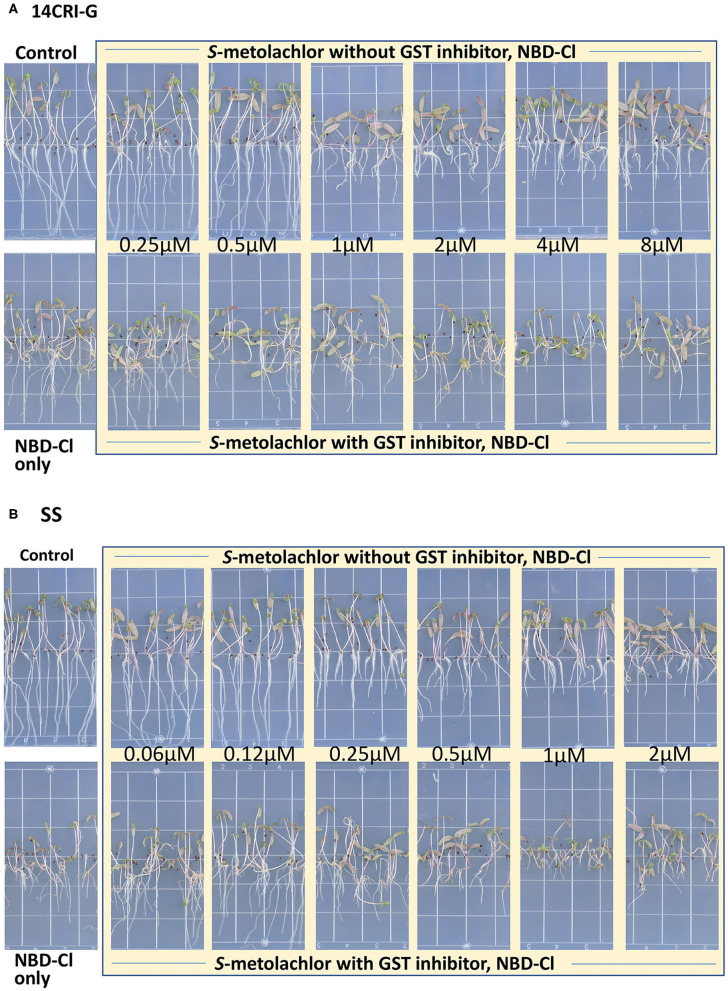
Effect of GST inhibitor, NBD-Cl, and *S*-metolachlor on seedling growth of *Amaranthus palmeri* in agar-based assays using a representative resistant [14CRI-G(A)] and susceptible [SS (B)] population. Root lengths were measured at 14 days of incubation in a growth chamber set at 30/28°C day/night temperature. The experiment was conducted twice. The upper and lower left panels of **(A)** and **(B)** are the non-treated and NBD-Cl treated checks of resistant and susceptible populations, respectively.

Sensitivity to *S*-metolachlor was observed as inhibition of root growth in agar-based assays (Figures 2, 3). Without NBD-Cl, the R and SS populations responded differently to *S*-metolachlor. The GR_50_ for SS, 15CRI-A, and 14CRI-G were 0.23, 0.92, and 0.74 μM, respectively (Figure 2). Based on the GR_50_, 15CRI-A, and 14CRI-G showed resistance levels of 4.1- and 3.6-fold in relation to SS (Table 2) in the agar medium. With NBD-Cl, the root growth of all populations was severely reduced, with further reduction as herbicide concentration increased. However, none of the regression models tested fit the data because of the small difference between the lower and upper asymptote. This was due to the significant toxicity of NBD-Cl alone. Regardless of the high growth inhibition by NBD-Cl alone, the data portrayed the combined effect of NBD-Cl and *S*-metolachlor on resistant populations (Figure 3). Without NDB-Cl, the highest dose tolerated by R populations was 0.5 μM *S*-metolachlor (Figure 3A). Above this dose, *S*-metolachlor caused severe inhibition of root growth. Adding NBD-Cl to 0.5 μM *S*-metolachlor resulted in greater root growth inhibition compared to NBD-Cl alone. This suggests that NBD-Cl suppressed the GSTs responsible for herbicide detoxification, making the R populations less resistant to *S*-metolachlor.

**Table 2:** Estimated GR_50_ for *S*-metolachlor tolerance in absence of NBD-Cl in *A. palmeri* populations using agar-based assay.

**Accession**	**GR50 (uM)**	**Fold change**
14CRI-G	0.750 (0.649–0.852)^*^	3.6
15CRI-A	0.929 (0.775–1.082)	4.1
SS	0.224 (0.185–0.263)	1.0

* *Values within brackets are the confidence intervals (p < 0.05) of the estimate*.

The highest dose tolerated by SS population was 0.12 μM (Figure 3B). The SS population response to 0.12 μM herbicide did not change in the presence of NBD-Cl; instead, lower concentration of *S*-metolachlor stimulated root growth. Also, comparing % root growth reduction with NBD-Cl alone across populations, SS showed the greatest inhibition (66%), followed by CRI-A (52%) and CRI-G (39%) (Figure 2). Considering that GST functions are not restricted to herbicide or xenobiotic detoxification, it appeared that the constitutive overexpression of some GSTs in the resistant populations afforded some protection from the phytotoxicity of NBD-Cl. The data also showed that *S*-metolachlor is more inhibitory to roots than other tissues of the seedling. This means that GST activity in roots is important in mitigating the phytotoxicity of the herbicide. Together, our data suggest that GSTs are key factors in *A. palmeri* resistance to *S*-metolachlor. A lower concentration of NBD-Cl than what was used in this study might allow better visualization of how much constitutive and inducible GST activity could impart resistance to *S*-metolachlor.

### Candidate *GST* Genes

The tau and phi class GST enzymes were chosen as putative candidate genes conferring resistance to *S*-metolachlor. To select the *GST* genes, we first identified the *GST* genes involved in VLCFA inhibitor tolerance or resistance in other species, such as *Z. mays* (corn), *L. rigidum* (rigid ryegrass) and *A. myosuroides* (blackgrass) from literature (Li et al., 2017a,b; Busi et al., 2018; Dücker et al., 2020). Access to recently sequenced and deposited *A. palmeri* genome allowed retrieval of putative homologs of such functionally related *GST* genes. As specific GSTs were elicited in response to safeners in *Arabidopsis* as a main component of molecular response (DeRidder and Goldsbrough, 2006), we also recovered its homologs in *A. palmeri*. The individual analysis of species-specific GSTs produced multiple candidate genes in *A. palmeri*. The topmost similar genes were selected using comparative analysis (BLAST). We selected five candidate genes for *S*-metolachlor detoxification. The identity of each selected candidate GSTs in *A. palmeri* and similarity with other known GSTs in different species are listed in Table 3. ApGSTU19 and ApGSTU18 were identified as the most probable homologs of tau class GSTs that were responsive to *S*-metolachlor or safener. ApGSTF2, ApGSTF8, and ApGSTF2like were included mainly in relation to safener inductivity.

**Table 3:** List of selected candidate GSTs in *A. palmeri* and its amino acid sequence similarities in other species.

**Putative candidate GSTs**			**Similarity with other species (name, protein sequence similarity)**			
***A. palmeri* locus name**	**GST name (adopted from *A. thaliana*)**	**GST class**	***A. thaliana***	***Z. mays***	***L. rigidum***	***A. myosuroides***
Ap.01g001210	ApGSTU19	Tau	AtGSTU19, 69%	ZmGST6, 59%	LrGST-1, 37%	AmGST1, 33%
				ZmGST5, 56%	LrGST-4, 40%	AmGST2, 51%!!break AmGST3, 57%
Ap.02g139000	ApGSTU18	Tau	AtGSTU18, 62%	ZmGST34, 46%	LrGST-1, 48%	AmGST1, 41%
					LrGST-4, 41%	AmGST2, 51%!!break AmGST3, 56%
Ap.06g223180	ApGSTF8	Phi	AtGSTF8, 50%	ZmGST12, 33%!!break ZmGST13, 32%	-	AmGST4, 25%
Ap.05g099350	ApGSTF2	Phi	AtGSTF2, 53%	-	-	-
Ap.05g099340	ApGSTF2like	Phi	AtGSTF2, 41%	-	-	-

*Protein sequences were aligned using the Clustal method and data are shown as percentage similarity*.

### Expression Profile of Candidate *GST*s

**Figure 4:**
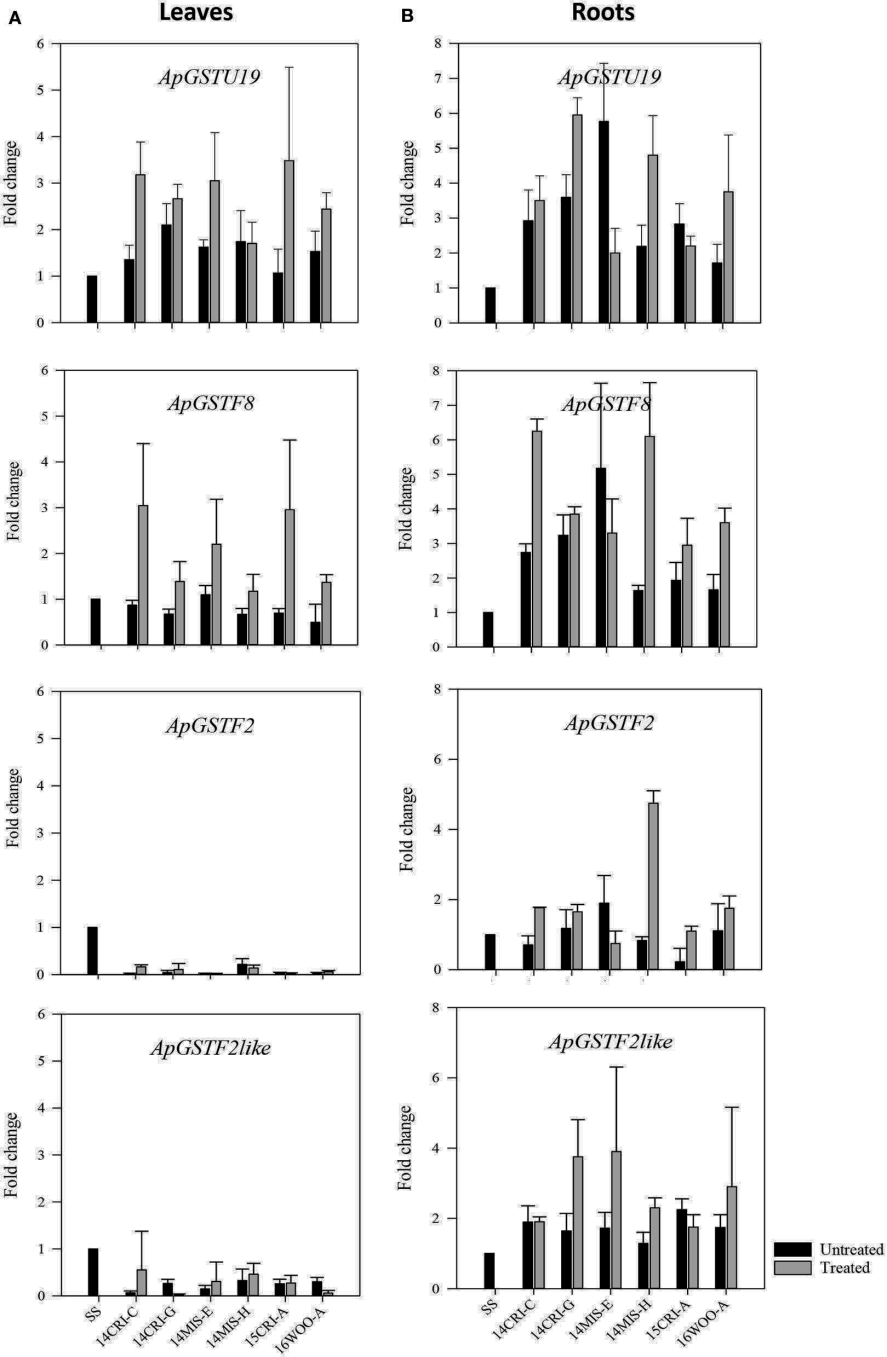
Expression profile of candidate *GST* genes in leaves **(A)** of *S*- metolachlor-resistant and -susceptible population of *A. palmeri* and roots **(B)**. Each bar represents the relative expression (fold change) of each gene in non-treated and treated resistant plants compared to non-treated susceptible plants (SS). Data are means ± SE of two independent experiments consisting of three biological replicates except roots of treated plants.

The expression of putative candidate *GST*s (Table 3) was measured in leaves and roots of survivors of 1x dose of *S*-metolachlor from resistant populations. This approach allowed the determination of the expression pattern of putative candidate *GST*s at the level of genetic transcription in different tissues, where herbicide detoxification could occur. The SS population was used as baseline. As *S*-metolachlor is a soil-applied herbicide, and our seeds were field-derived, it was impossible to obtain non-treated R plants. We could only recover confirmed R plants (the survivors); therefore, non-treated SS plants were used as control to estimate the constitutive and inductive expression level of candidate genes in R plants. Four genes, *ApGSTU19, ApGSTF2, ApGSTF8*, and *ApGSTF2like* were differentially regulated between survivors and SS control plants (Figure 4). The expression of *ApGSTU18* did not differ significantly between R and SS plants in roots and leaves (Supplementary Figure 2). The expression of *ApGSTU19* and *ApGSTF8* were constitutively upregulated in the roots of R plants. *ApGSTF8* was further upregulated three- to six-fold higher in roots in response to *S*-metolachlor in R populations. The expression of *ApGSTU19* varied from two- to six-fold in treated R populations. Interestingly, *ApGSTF8* and *ApGSTU19* were not constitutively upregulated in leaves. Survivors from 14MIS-E, 15CRI-A, and 14CRI-C showed two- to three-fold induction of *ApGSTF8* in leaves, whereas other populations did not show significant induction. Slight upregulation of *ApGSTU19* was also observed in leaves of survivors. The expression level of *ApGSTF2* and *ApGSTF2like* gene was higher in roots than leaves. These genes were not enriched in leaves of R plants; instead, its expression remained lower in leaves of R plants across populations compared to non-treated SS. *ApGSTF2* and *ApGSTF2like* were enriched approximately two-fold in roots of the majority of R plants upon *S*-metolachlor treatment. Survivors from 14MIS-H showed the highest induction (~4-fold) of *ApGSTF2*, while those of 14MIS-E, 14CRI-G and 16WOO-A showed three to four-fold induction of *ApGSTF2like* gene in roots upon herbicide treatment. Overall, the comparison of tissue-specific transcript abundance showed higher expression of selected candidate genes in roots. Also, the expression level of candidate genes was not directly correlated with the level of resistance.

### Phylogenetic Analysis and Substrate Binding Site Comparison

**Figure 5:**
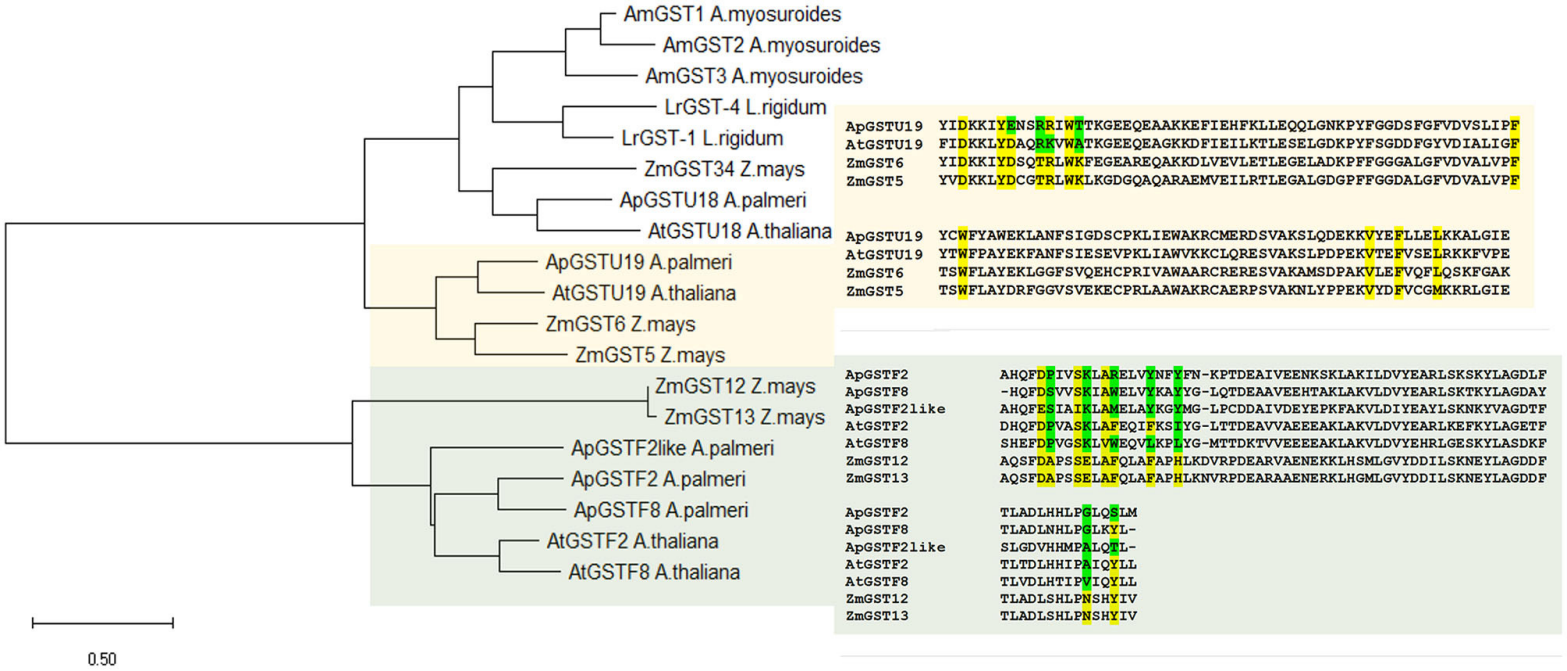
Phylogenetic and active site analysis of selected GSTs from different species. The selected GSTs were analyzed from *Arabidopsis thaliana, Zea m*ays, *Lolium rigidum, Aloperucrus myosuroides*, and *Amaranthus palmeri*. The evolutionary history was inferred using the Maximum Likelihood method and JTT matrix-based model (Jones et al., 1992). The tree is drawn to scale, with branch lengths measured in the number of substitutions per site. This analysis involved 19 amino acid sequences, and the final dataset was composed of 285 positions. Evolutionary analyses were conducted in MEGA X (Kumar et al., 2018). Active site in candidate ApGSTU19 and ApGSTF8 was identified by comparing the sequence with *Z. mays*. Highlighted in yellow is the conserved and green is the polymorphic compared to *Z. mays* active site residue. Multiple sequence alignment was carried out using uniport align tool using ApGSTU19, ApGSTF8, ApGSTF2, and ApGSTF2like from *A. palmeri*, AtGSTU19 (AT1G78380), AtGSTF2 (AT4G02520), and AtGSTF8 (AT1G02920) in *A. thaliana* and ZmGST5 (CAA73369), ZmGST6 (CAB38120), ZMGST12 (AAG34820), and ZmGST13 in *Z. mays* (AAG34821).

Phylogenetic analysis of selected candidate genes indicated that these genes are evolutionarily related across different species and could be functionally related (Figure 5). Tau class GST enzyme, ApGSTU19, is closely related to AtGST19 in *Arabidopsis* and ZmGST6 in *Z. mays*. Similarly, all phi GST enzymes included in this study are highly similar to each other. ApGSTF8 in *A. palmeri* is highly similar to ZmGST12 and ZmGST13 in *Z. mays* (Figure 3).

The annotation of substrate-binding site or H-site in known GST enzymes provides more detailed insights regarding critical amino acids involved in specific herbicide conjugation. Only proteins with high % sequence similarity between *A. palmeri* and other species from Table 3 were selected for comparison of H-site. Since the *Z. mays* GST enzymes are best characterized with respect to chloroacetanilide herbicide detoxification, its H-site was used as reference to compare the similarity with candidate genes. First, the amino acid sequences of ApGSTU19 and the functionally related AtGSTU19, ZmGST6, and ZmGST5 enzymes were aligned and the H-site was annotated using information available at the NCBI Conserved Domain Database (CDD) (Lu et al., 2020). To rule out the possible contribution of any coding sequence mutation in ApGSU19 to *A. palmeri* resistance to *S*-metolachlor, nearly full-length sequence was obtained using survivors from 15CRI-A to 14CRI-G populations. There were no changes in the H-site residues compared to the reference sequence (Supplementary Figure 3). Analysis of the H-site of ApGSTU19 showed that the majority of residues are conserved between ApGSTU19 in *A. palmeri* and ZmGST6 and ZmGST5 in *Z. mays* (Supplementary Figure 5). Thus, we can predict that their biological function would be similar. The H-site of ApGSTU18 was significantly different from those of related enzymes, especially that of ZmGST34 (Supplementary Figure 4), which is involved in most chloroacetanilide herbicide detoxification (Li et al., 2017c). Also, the H-site of all selected phi enzymes was very different from those of ZmGST12 and ZmGST13 (Figure 5).

## Discussion

VLCFA-inhibiting herbicides are used to control a broad spectrum of broadleaf and grass weeds in various crops. Seven weed species; *L. rigidum, A. myosuroides, A. palmeri, Amaranthus tuberculatus, Avena fatua, Echinochloa crus-galli* var. *crus-galli*, and *Lolium perenn*e ssp. *Multiflorum* have now evolved resistance to VLCFA inhibitors (WSSA-Group 15) around the world (Heap, 2021). This study aimed to understand the recent evolution of *A. palmeri* resistance to VLCFA inhibitors. Resistance to VLCFA inhibitors has evolved slowly with time, despite the sustained, high-volume usage of this group of herbicides across a large number of crops across decades. This is a testament to the difficulty of overcoming the phytotoxic effect of this type of herbicide action. The range of resistance level found in this study was wide (3-to 29-fold). For a soil-applied herbicide, this is primarily a reflection of emergence reduction, and the degree of stunting of emerged seedlings. Thus, we can equate low resistance index to less reduction in plant population. As with foliar herbicides, low frequency of resistant individuals produces low resistance indices, indicative of an early stage of resistance evolution. Populations with high resistance index are more purified, having gone through multiple cycles of reproduction of R plants and concomitant selection with the same herbicide SOA. It can also be hypothesized that a high resistance index is a latent effect of preexisting NTSR mechanisms selected by other primary herbicide selectors. In the case of Arkansas populations, this includes ALS- and PPO inhibitors. Except 16WOO-A, all other resistant populations are reported to have reduced sensitivity to multiple herbicides with different SOAs in a previous study (Salas-Perez et al., 2017). All the resistant populations used in this study showed high resistance to sub-lethal dose of *S*-metolachlor (Supplementary Figure 1), which is indicative of elevated baseline protection, characteristic of NTSR evolution.

Upon application of alachlor and *S*-metolachlor in crops like corn, these herbicides are found as nontoxic GSH conjugates, thus, providing tolerance to these herbicides (Shimabukuro et al., 1971; Cottingham and Hatzios, 1992; Rossini et al., 1996). These early studies suggested that enhanced metabolism driven by GSTs is the mechanism of tolerance to chloroacetanilide. Recent research on *L. Rigidum* and *A. myosuroides* resistance to pyroxasulfone and flufenacet also indicated detoxification by GSTs as the primary mechanism of resistance (Busi et al., 2018; Dücker et al., 2020). The effect of GST inhibitor on the activity of *S*-metolachlor on *A. palmeri* in our study and that of Brabham et al. (2019) indicate that GSTs endow resistance to *S*-metolachlor in *A. palmeri*. The involvement of GSTs was supported by gene expression data of *A. palmeri* GSTs in R and S plants. The four candidate *GST* genes, which showed differential response between R and S plants showed higher expression in roots than in shoots, indicating that seedlings survive because a substantial amount of the herbicide is inactivated by GSTs as soon as it is absorbed by the roots (Figure 4). This was achieved by constitutive upregulation of GSTs in the roots of R plants and further induction of these GSTs in the presence of *S*-metolachlor. The suppression of GST activity by NBD-Cl, reduced the root growth more than it did the shoot growth (Figure 2). In S plants where GSTs were not constitutively upregulated, NBD-Cl did not affect the activity of *S*-metolachlor. Collectively, these results suggest that roots are the primary site where *S*-metolachlor detoxification by GSTs occur. This makes sense for a soil-applied herbicide with this SOA and corresponding mechanism of natural plant protection. Our findings are consistent with previous research correlating enhanced GST activity in roots of maize cultivar with tolerance to chloroacetanilide herbicides (Sari-Gorla et al., 1993; Li et al., 2017b).

Safeners are agrochemicals that physiologically increase herbicide protection by enhancing the production of selective or distinct GSTs in crops (Hatzios and Wu, 1996; DeRidder et al., 2002; Scarponi et al., 2006). Much work has been done to understand specific GSTs that are responsive to safeners using *Arabidopsis* as a model. In our study we chose GSTs responsive to safeners, benoxacor, and fluxofenim, as candidates for *S*-metolachlor degradation as these safeners are commercially used to safen grass crops from metolachlor (Davies and Caseley, 1999). These candidate genes sourced from multiple species showed evolutionary relationship with *A. palmeri* GSTs (Figure 5); therefore, we speculated that the *A. palmeri* homologs would retain a similar biological function in response to *S*-metolachlor, safening the weed from the herbicide.

*ApGSTU19*, which is a tau class GST, was differentially expressed in all R populations compared to SS population (Figure 4). *ApGSTU19* showed high similarity to the *AtGSTU19* in *Arabidopsis*, which was induced in response to several safeners (DeRidder et al., 2002; DeRidder and Goldsbrough, 2006; Skipsey et al., 2011). *AtGSTU19* was highly induced by benoxacor and fluxofenim, and was able to conjugate alachlor, acetochlor, and *S*-metolachlor to GSH *in vitro* (DeRidder et al., 2002). The GST enzymes contain an N-terminal thioredoxin-fold domain and a C-terminal alpha helical domain, with H-site located in a cleft between the two domains. GSH binds to the N-terminal domain called G-site, while the substrate occupies a pocket in the C-terminal domain known as H-site, together it is known as active site of GSTs. The G-site is conserved, but H-site varies between different GSTs. Active site comprising multiple overlapping hydrophobic residues within H-site allows a wide range of substrates to bind, which allows GSTs to act on diverse xenobiotics (Cummins et al., 2011). ApGSTU19 also showed high similarity, in sequence and H-site, to ZmGST5 and ZmGST6 in maize (Figure 5), indicating the same ability to catalyze the conjugation of GSH to *S*-metolachlor in resistant *A. palmeri* plants. Li et al. (2017a) demonstrated that *ZmGST5* and *ZmGST6* are one of the highly expressed core genes among the other *GSTs* that were found to be responsible for differential tolerance to metolachlor in tolerant maize cultivar. *In vitro* studies using recombinant maize ZmGST5 showed specificity of this GST toward *S*-metolachlor (Dixon et al., 1997, 1998). Thus, we propose that *ApGSTU19* is involved in detoxification of *S*-metolachlor in *A. palmeri*.

*ApGSTU19* and *ApGSTF8* were constitutively upregulated in roots, but not in leaves, and their abundance increased in response to *S*-metolachlor in R populations. This indicates tissue-specificity geared toward protection from soil-applied herbicides, elevated baseline protection, and increased protection when challenged with herbicide. High expression of *AtGSTU19* and *AtGSTF8* was also predominant in roots and suspension culture in response to different safeners in *Arabidospis* (DeRidder et al., 2002; DeRidder and Goldsbrough, 2006; Skipsey et al., 2011). Other researchers reported similar root-specific expression profile of *ZmGST6* and other phi genes, *ZmGST12* and *ZmGST13*, accounting for crop tolerance to *S*-metolachlor (Li et al., 2017b). *ApGSTF8*, a phi class GST, was consistently the most differentially responsive among all candidate genes in roots of all R plants (Figure 4). *ApGSTF8* was highly similar to *AtGSTF8* (Table 3), which is known to be highly responsive to the safener benoxacor (DeRidder et al., 2002). In this comprehensive *Arabidopsis* study, which showed the induction of multiple GST enzymes by different safeners, the expression profile of AtGSTF8 differed from all other *GST* genes tested, showing the greatest induction by benoxacor. Benoxacor is typically added to *S*-metolachlor for use in corn to reduce injury. ApGSTF8 was also closely related to ZmGST12 and ZmGST13 enzymes (Figure 5), which was highly upregulated in roots of maize cultivar with differential tolerance to *S*-metolachlor (Li et al., 2017b), suggesting that ApGSTF8 may contribute to *S-*metolachlor detoxification in *A. palmeri*. The H-site analysis of ApGSTF8 enzyme did not show high similarity (Figure 5) to other functionally related GSTs, but its consistent differential upregulation in roots of all R plants strongly suggests involvement in *S*-metolachlor detoxification. Other phi class candidate genes, *ApGSTF2* and *ApGSTF2like* are closely related to *ApGSTF8*, but their level of expression was different among R plants from different R populations (Figure 4). The R populations have evolved independently of each other, considering their geographical separation; therefore, could harbor different regulation patterns of GST isozymes. AtGSTF2 is greatly induced by fluxofenim in *Arabidopsis*; thus, we expect ApGSTF2 and ApGSTF2like isozymes to react with *S*-metolachlor as well, protecting R plants. Position of *ApGSTF2* and *ApGSTF2like* gene is located adjacent to each other within the DNA sequence of *A. palmeri* genome. Our data suggests that *ApGSTF2* and *ApGSTF2like* may not be cotranscribed and are transcribed at different levels despite their close genetic linkage.

The expression of *ApGSTU18*, also a tau class GST, was not different in R plants regardless of being 50% similar to *ApGSTU19*. ApGSTU18 was chosen as a candidate GST based on its sequence similarity to LrGST1, AmGST2, and AmGST3, which were identified to confer metabolic resistance to pyroxasulfone and flufenacet in *L. rigidum* and *A. myosuroides*, respectively (Table 3). Sequence comparison also showed that ApGSTU18 was 46% similar to ZmGST34, which was found to be differentially regulated in maize cultivar tolerant to *S*-metolachlor. Transgenic *Arabidopsis* plants overexpressing *ZmGST34* had increased tolerance to most chloroacetanilide herbicides (Li et al., 2017c). Active site comparison between ApGSTU18 and ZmGST34 showed minimal identity (Supplementary Figure 4). Therefore, it is possible that the differential response of *ApGSTU18* and *ApGSTU19* to *S-*metolachlor in *A. palmeri* may lie in the difference between the topology of the substrate binding site.

The co-ordinated up-regulation of genes encoding candidate GST enzymes showed substantial similarity with those induced by safeners in *Arabidopsis*. Although treatment with various safeners failed to impart tolerance to chloroacetanilide herbicides in *Arabidopsis* (DeRidder and Goldsbrough, 2006), eliciting the expected safener response in resistant *A. palmeri* plants indicates strong association between the function of these candidate GSTs and metolachlor detoxification. Structural difference among GSTs certainly alters reactivity to *S*-metolachlor and affects resistance level. Differential resistance level to *S*-metolachlor in *A. palmeri* could also be due to modifications in components of phase III detoxification. Although all candidate GSTs are differentially regulated in R plants compared to S plants, the specificity of an individual GST toward *S-*metolachlor should be tested either using heterologous expression system or *in vitro* assay to show, unequivocally, that these GSTs are responsible for *S*-metolachlor detoxification in *A. palmeri*. Transcriptome and metabolism studies are warranted.

## Data Availability Statement

The original contributions presented in the study are included in the article/Supplementary Materials, further inquiries can be directed to the corresponding author/s.

## Author Contributions

GR designed and conducted the candidate GST analysis, performed the molecular biology experiments, analyzed the data, and wrote the manuscript. MN performed the herbicide dose-response and GST-inhibitor assays and participated in the manuscript preparation. LB contributed to the molecular biology experiments. RS-P participated in field sampling of Palmer amaranth populations with NR-B. RS-P conducted the first general screening for *S*-metolachlor resistance and characterized the resistance profile of populations used in this study. NR-B conceived the whole Palmer amaranth project, obtained funding for the project, organized and led the collection of Palmer amaranth samples and field histories, conceptualized the phenotyping and molecular biology experiments, directed the research implementation, and worked with GR in manuscript writing and revisions. All authors contributed to the article and approved the submitted version.

## Conflict of Interest

The authors declare that the research was conducted in the absence of any commercial or financial relationships that could be construed as a potential conflict of interest.
