# Supplementary material for: Mechanism of Resistance to S-metolachlor in Palmer amaranth
Source: Front Plant Sci. 2021 Mar 12;12:652581. doi: 10.3389/fpls.2021.652581 (PMC7994610; doi:10.3389/fpls.2021.652581)
Supplement: Supplementary file 1 [file Data_Sheet_1.PDF]

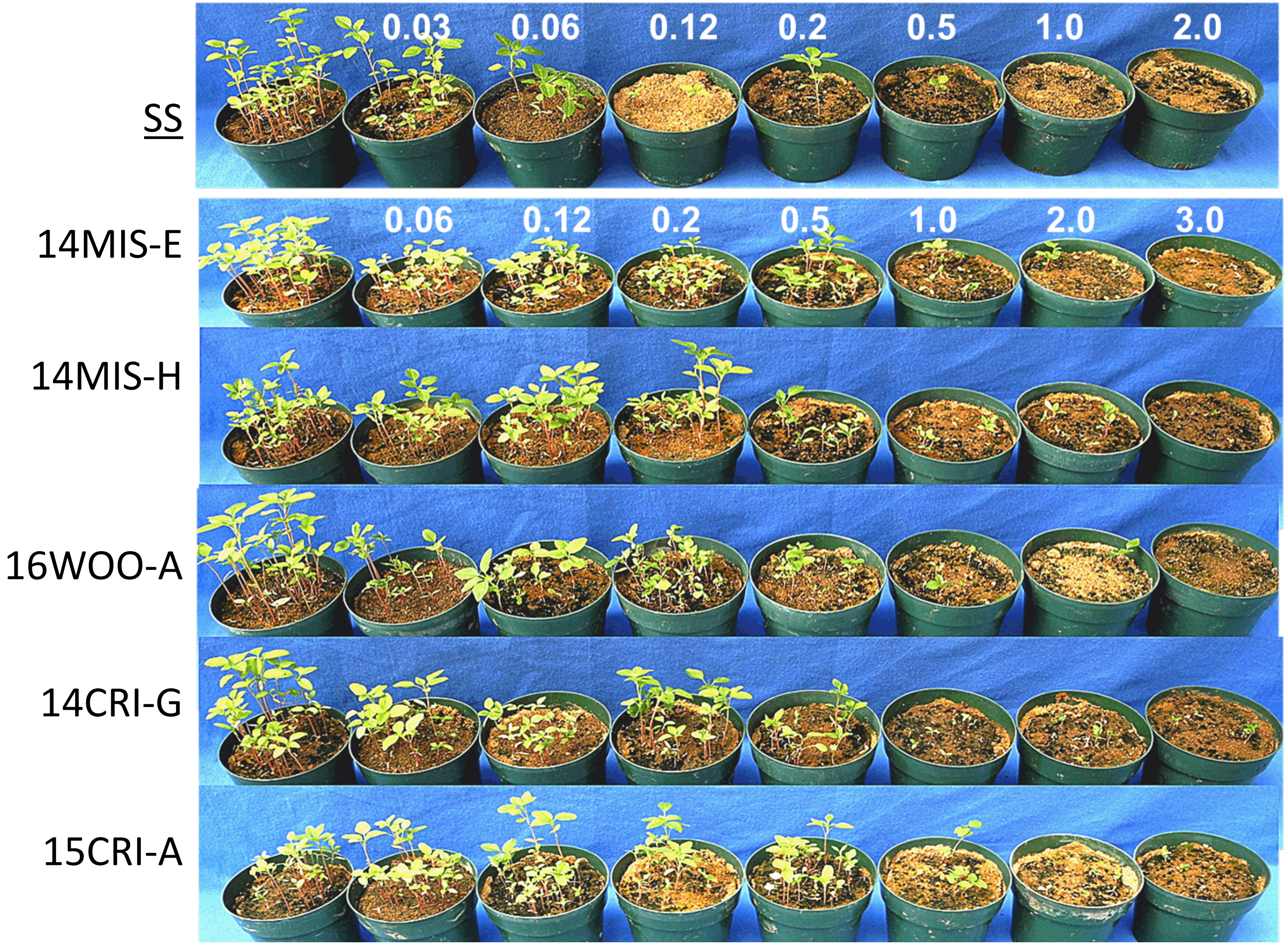

Supplementary Figure 1. *S*-metolachlor-resistant and -susceptible *Amaranthus palmeri* populations in a greenhouse dose-response experiment. SS = susceptible standard; all others are resistant. Doses for the resistant populations were: 0.06x, 0.12x, 0.2x, 0.5x, 1x, 2x and 3x. The SS was treated with 0.03x, 0.06x, 0.12x, 0.2x, 0.5x, 1x and 2x. The 1x dose was 1.1 kg ai ha^{-1}. Photos were taken 21 d after planting. The first pot of each row was nontreated check.

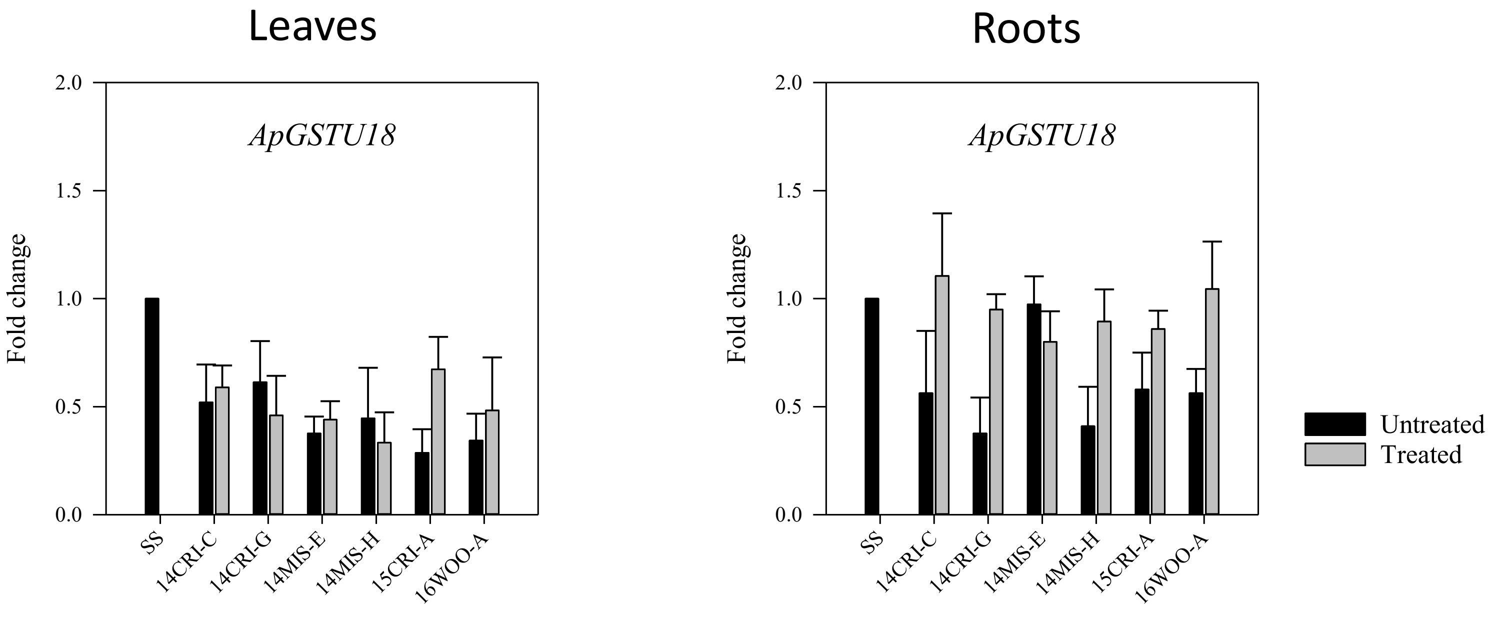

Supplementary Figure 2. Expression profile of *ApGSTU18* gene in *S*-metolachlor-resistant and -susceptible (SS) populations of *Amaranthus palmeri* in leaves and roots. Each bar represents the relative expression (fold change) of *ApGSTU18* in nontreated and treated samples from resistant populations compared to nontreated SS. Expression analysis was carried out by real-time qPCR. Data are means ± SE of two independent experiments consisting three biological replicates except treated root plants where two biological replicates were used. Expression was normalized using *B-tubulin* and *elongation factor1α* .

```
ApGSTU19_Ref       MADEVVLLDFWVSMFGMRVRIALAEKGVKYEYKEQDLRNKSDLLLKMNPVHKKIPVLIHN 60
ApGSTU19_15CRI-A   ---------------------HSPEKDVKYEYKEQDLRNKSDLLLKMNPVHKKIPVLIHN 39
ApGSTU19_14CRI-G   ---------------------ALAEKDVKYEYKEQDLRNKSDLLLKMNPVHKKIPVLIHN 39
                                           **.*********************************

ApGSTU19_Ref       NKPVCESTIIVQYIDEVWNDKNPLMPSDPYQRAQARFWADYIDKKIYENSRRIWTTKGEE 120
ApGSTU19_15CRI-A   NKPVCESTIIVQYIDEVWNDKNPLMPSDPYQRAQARFWADYIDKKIYENSRRIWTTKGEE 99
ApGSTU19_14CRI-G   NKPVCESTIIVQYIDEVWNDKNPLMPSDPYQRAQARFWADYIDKKIYENSRRIWTTKGEE 99
                   ************************************************************

ApGSTU19_Ref       QEAAKKEFIEHFKLLEQQLGNKPYFGGDSFGFVDVSLIPFYCWFYAWEKLANFSIGDSCP 180
ApGSTU19_15CRI-A   QEAAKKEFIEHFKLLEQQLGNKPYFGGDSFGFVDVSLIPFYCWFYAWEKLANFSIGDSCP 159
ApGSTU19_14CRI-G   QEAAKKEFIEHFKLLEQQLGNKPYFGGDSFGFVDVSLIPFYCWFYAWEKLANFSIGDSCP 159
                   ************************************************************

ApGSTU19_Ref       KLIEWAKRCMERDSVAKSLQDEKKVYEFLLELKKALGIE 219
ApGSTU19_15CRI-A   KLIEWAKRCMERDSVAKSLQDEKKVYEFLLELKKALGI- 197
ApGSTU19_14CRI-G   KLIEWAKRCMERDSVAKSLQDEK---------------- 182
                   ***********************
```

Supplementary Figure 3. Sequence alignments of ApGSTU19 between resistant populations. Alignment was performed between deduced protein sequence from sequencing results of two individuals from 14CRI-G and 15CRI-A populations and reference *A. palmeri* gene (Ap.01g001210) using Uniport align tool. The active site was determined using CD-search tool available at NCBI and manually annotated (highlighted in yellow). Analysis showed no difference in active site residues between reference and resistant ApGSTU19 peptide.

```
ApGSTU18    AAYIDDKWFPSLNGMRKAETEEEKVAAINEVKEGLLVLEDAFEKCSKGKPYFNGDHIGYL
AtGSTU18    AAYIDDQWFISVRSILTAQGDEEKKAAIAQVEERTKLLEKAFNDCSQGKPFFNGDHIGYL
ZmGST34     AQYVDDKMHPAIR-VLKGTYDGDKEQAAGQLSAALQLLEEAFAQLGQGKRYFGGDSVGYL
AmGST2      AAYIDDKLIVAWRQAFSGKREEDKSEGTKQMFAALDILEEALRECSKGHGYFGGESVGLV
AmGST3      AAYIDDKLLASWLQAARGKTDEEKTEGLKQTFVAVETMEAAFKTCSKGKPFFGGDSVGYL
LrGST-1     AVYVDDKFFPAWLGILRAETEEERAKKMSETAAVVEQLEAALAQCSNGKAFFSGDSVGYL

ApGSTU18    DIALGSYLGWLRVVEKMNNVVLLDQEKTPKLCAWAQNFCGDDAVKDYMPETDKLIEFAKI
AtGSTU18    DIALGSFLGWWRVVELDANHKFLDETKTPSLVKWAERFCDDPAVKPIMPEITKLAEFARK
ZmGST34     DIALVSHVGWVKAVEKIAGVTLLDEAKVPNLVAWADRLCAHPAVVDAIPDADKFVEFSVT
AmGST2      DVWLGSLLSWLKASAVNSGIQIFDPIKTPLLTAWMERFSELDSAKAALPDVDRVIEFGKM
AmGST3      DVALGALVA---------------------------------------------------
LrGST-1     DIAVGCNLFWLDAMRKMFGVVVIDAARTPVLAAWADRFRESDVGKEVLPDGDIAVEYAKK
```

Supplementary Figure 4. Active site comparison between ApGSTU18 and related proteins. Multiple sequence alignment was done using uniport align tool. The conserved binding sites were subsequently annotated manually in ApGSTU18 from *A. palmeri* (Ap.02g139000), AtGSTU18 in *A. thaliana* (AT1G10360), ZmGST34 in *Z. mays* (AAG34842), AmGST2 in *A. myosuroides* (Alomy042368), AmGST3 in *A. myosuroides* (Alomy056271) and LrGST-1 in *L. rigidum*

Table S1: RNA primers used for the qRT-PCR gene expression assay of selected candidate genes and full-length sequencing of *ApGSTU19*

**Gene**	**Fwd/ Rev**	**Sequence (5' to 3')**
*ApGSTU19(*qPCR)	Forward	GCCATATTTGGAGGGGATT
	Reverse	TTGGCTAGTTTTTCCCAAGC
ApGSTU18(qPCR)	Forward	AGTCGAGGCTCATCAATTCG
	Reverse	GGTTTGCAAGCCGTAATAGG
ApGSTF2(qPCR)	Forward	CTCGCACGAGAGCTTGTTTA
	Reverse	CGATACCAAGTGCCTTCTTCA
ApGSTF2-like(qPCR)	Forward	CCAACAAAGGGAACCATCT
	Reverse	GCCTTCGTAAGTGTATGCTAT
ApGSTF8(qPCR)	Forward	AGTCGAGGCTCATCAATTCG
	Reverse	GGTTTGCAAGCCGTAATAGG
ApGSTU19(full-length)	Forward	GCACTCGCCGAAAAAGATG
	Reverse	CGATACCAAGTGCCTTCTTCA
ApEF1α	Forward	TGAGGCTGGTATCTCCAAGG
	Reverse	TGGTGGCATCCATCTTGTTA
